# How Citation Boosts Promote Scientific Paradigm Shifts and Nobel Prizes

**DOI:** 10.1371/journal.pone.0018975

**Published:** 2011-05-04

**Authors:** Amin Mazloumian, Young-Ho Eom, Dirk Helbing, Sergi Lozano, Santo Fortunato

**Affiliations:** 1 ETH Zürich, Zürich, Switzerland; 2 Complex Networks and Systems Lagrange Laboratory, Institute for Scientific Interchange (ISI), Torino, Italy; University of Zaragoza, Spain

## Abstract

Nobel Prizes are commonly seen to be among the most prestigious achievements of our times. Based on mining several million citations, we quantitatively analyze the processes driving paradigm shifts in science. We find that groundbreaking discoveries of Nobel Prize Laureates and other famous scientists are not only acknowledged by many citations of their landmark papers. Surprisingly, they also boost the citation rates of their previous publications. Given that innovations must outcompete the rich-gets-richer effect for scientific citations, it turns out that they can make their way only through citation cascades. A quantitative analysis reveals how and why they happen. Science appears to behave like a self-organized critical system, in which citation cascades of all sizes occur, from continuous scientific progress all the way up to scientific revolutions, which change the way we see our world. Measuring the “boosting effect” of landmark papers, our analysis reveals how new ideas and new players can make their way and finally triumph in a world dominated by established paradigms. The underlying “boost factor” is also useful to discover scientific breakthroughs and talents much earlier than through classical citation analysis, which by now has become a widespread method to measure scientific excellence, influencing scientific careers and the distribution of research funds. Our findings reveal patterns of collective social behavior, which are also interesting from an attention economics perspective. Understanding the origin of scientific authority may therefore ultimately help to explain how social influence comes about and why the value of goods depends so strongly on the attention they attract.

## Introduction

Ground-breaking papers are extreme events [1] in science. They can transform the way in which researchers do science in terms of the subjects they choose, the methods they use, and the way they present their results. The related spreading of ideas has been described as an epidemic percolation process in a social network [2]. However, the impact of most innovations is limited. There are only a few ideas, which gain attention all over the world and across disciplinary boundaries [3]. Typical examples are elementary particle physics, the theory of evolution, superconductivity, neural networks, chaos theory, systems biology, nanoscience, or network theory.

It is still a puzzle, however, how a new idea and its proponent can be successful, given that they must beat the rich-gets-richer dynamics of already established ideas and scientists. According to the Matthew effect [4]–[7], famous scientists receive an amount of credit that may sometimes appear disproportionate to their actual contributions, to the detriment of younger or less known scholars. This implies a great authority of a small number of scientists, which is reflected by the big attention received by their work and ideas, and of the scholars working with them [8].

**Figure 1:**
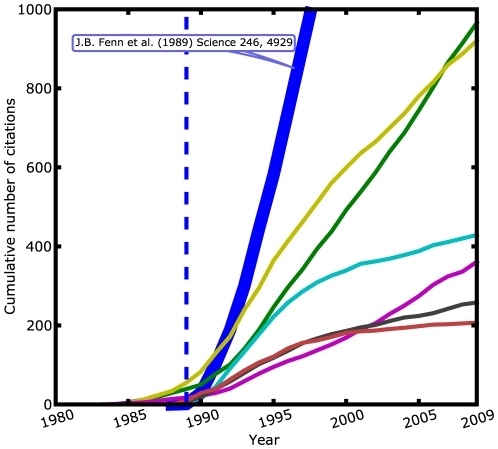
Illustration of the boosting effect. Typical citation trajectories of papers, here for Nobel Prize Laureate John Bennett Fenn, who received the award in chemistry in 2002 for the development of the electrospray ionization technique used to analyze biological macromolecules. The original article, entitled *Electrospray ionization for mass spectrometry of large biomolecules*, coauthored by M. Mann, C. K. Meng, S. F. Wong and C. M. Whitehouse, was published in *Science* in 1989 and is the most cited work of Fenn, with currently over 3,000 citations. The diagram reports the growth in time of the total number of citations received by this landmark paper (blue solid line) and by six older papers. The diagram indicates that the number of citations of the landmark paper has literally exploded in the first years after its appearance. However, after its publication in 1989, a number of other papers also enjoyed a much higher citation rate. Thus, a sizeable part of previous scientific work has reached a big impact after the publication of the landmark paper. We found that the occurrence of this boosting effect is characteristic for successful scientific careers.

Therefore, how can a previously unknown scientist establish at all a high scientific reputation and authority, if those who get a lot of citations receive even more over time? Here we shed light on this puzzle. The following results for 124 Nobel Prize Laureates in chemistry, economics, medicine and physics suggest that innovators can gain reputation and innovations can successfully spread, mainly *because* a scientist's body of work overall enjoys a greater impact after the publication of a landmark paper. Not only do colleagues notice the ground-breaking paper, but the latter also attracts the attention to older publications of the same author (see Fig. 1). Consequently, *future* papers have an impact on *past* papers, as their relevance is newly weighted.

We focus here on citations as indicator of scientific impact [9]–[13], studying data from the ISI Web of Science, but the use of click streams [14] would be conceivable as well. It is well-known that the relative number of citations correlates with research quality [15]–[17]. Citations are now regularly used in university rankings [18], in academic recruitments and for the distribution of funds among scholars and scientific institutions [19].

## Results

**Figure 2:**
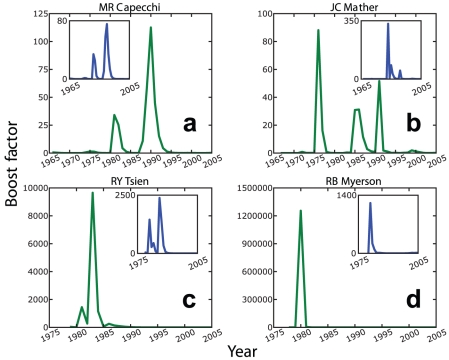
Typical time evolutions of the boost factor. Temporal dependence of 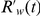 for Nobel Laureates [here for (a) Mario R. Capecchi (Medicine, 2007), (b) John C. Mather (Physics, 2006), (c) Roger Y. Tsien (Chemistry, 2008) and (d) Roger B. Myerson (Economics, 2007)]. Sharp peaks indicate citation boosts in favor of older papers, triggered by the publication and recognition of a landmark paper. Insets: The peaks even persist (though somewhat smaller), if in the determination of the citation counts 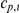, the landmark paper is skipped (which is defined as the paper that produces the largest reduction in the peak size, when excluded from the computation of the boost factor). We conclude that the observed citation boosts are mostly due to a collective effect involving several publications rather than due to the high citation rate of the landmark paper itself.

**Figure 3:**
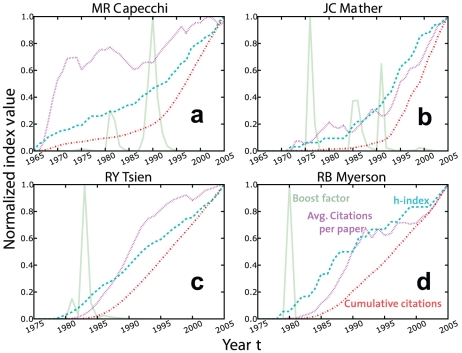
Dynamics of the boost factor 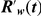 versus traditional citation variables. Each panel displays the time histories of four variables: the boost factor 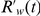, the average number of citations per paper 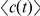, the cumulative number of citations 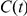, and the 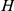-index earned until year 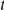 [21]. The panels refer to the same Nobel Laureates as displayed in Fig. 2. The classical indices have relatively smooth profiles, i.e. they are not very sensitive to extreme events in the life of a scientist like the publication of landmark papers. An advantage of the boost factor is that its peaks allow one to identify scientific breakthroughs earlier.

**Figure 4:**
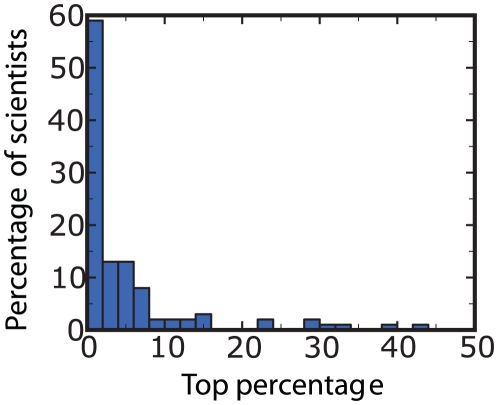
Correlation between papers and the local maxima (“peaks”) of 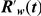. We first determined the ranks of all papers of an author based on the total number of citations received until the year 2009 inclusively. We then determined the rank of that particular publication, which had the greatest contribution to the peak. This was done by measuring the reduction in the height of the peak, when the paper was excluded from the calculation of the boost factor (as in the insets of Fig. 2). The distribution of the ranks of “landmark papers” is dominated by low values, implying that they are indeed among the top publications of their authors.

We evaluated data for 124 Nobel Prize Laureates that were awarded in the last two decades (1990-2009), which include an impressive number of about 2 million citations. For all of them and other internationally established experts as well, we find peaks in the changes of their citation rates (Figs. 2 and 3). Moreover, it is always possible to attribute to these peaks landmark papers (Fig. 4), which have reached hundreds of citations over the period of a decade. Such landmark papers are rare even in the lives of the most excellent scientists, but some authors have several such peaks.

Technically, we detect a groundbreaking article 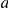 published at time 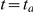 by comparing the citation rates before and after 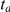 for the earlier papers. The analysis proceeds as follows: Given a year 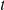 and a time window 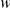, we take all papers of the studied author that were published since the beginning of his/her career until year 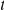. The citation rate 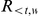 measures the average number of citations received per paper per year in the period from 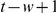 to 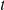. Similarly, the citation rate 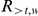 measures the average number of citations received by the same publications per paper per year between 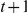 and 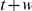 (or 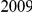, if 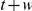 exceeds 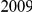). The ratio 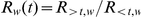, which we call the “boost factor”, is a variable that detects critical events in the life of a scientist: sudden increases in the citation rates (as illustrated by Fig. 1) show up as peaks in the time-dependent plot of 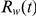.

In our analysis we used the generalized boost factor 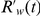, which reduces the influence of random variations in the citation rates (see Materials and Methods).

Figure 2 shows typical plots of the boost factors 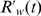 of four Nobel Prize Laureates. Interestingly, peaks are even found, when those papers, which mostly contribute to them, are *excluded* from the analysis (see insets of Fig. 2). That is, the observed increases in the citation rates are not just due to the landmark papers themselves, but rather to a collective effect, namely an increase in the citation rates of *previously* published papers. This results from the greater visibility that the body of work of the corresponding scientist receives after the publication of a landmark paper and establishes an increased scientific impact (“authority”). From the perspective of attention economics [20], it may be interpreted as a herding effect resulting from the way in which relevant information is collectively discovered in an information-rich environment. Interestingly, we have found that older papers receiving a boost are not always works related to the topic of the landmark paper.

Traditional citation analysis does not reveal such crucial events in the life of a scientist very well. Figure 3 shows the time history of three classical citation indices: the average number of citations per paper 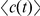, the cumulative number 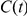 of citations, and the Hirsch index [21] (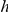-index) 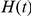 in year 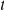. For comparison, the evolution of the boost factor 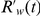 is depicted as well. All indices were divided by their maximum value, in order to normalize them and to use the same scale for all. The profiles of the classical indices are rather smooth in most cases, and it is often very hard to see any significant effects of landmark papers. However, this is not surprising, as the boost factor is designed to capture abrupt variations in the citation rates, while both 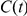 and 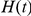 reflect the overall production of a scientist and are therefore less sensitive to extreme events.

**Figure 5:**
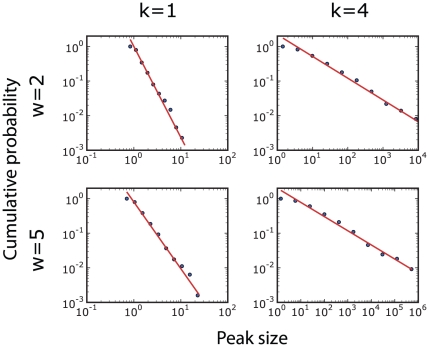
Cumulative probability distribution of peak heights in the boost factor curves of Nobel Prize Laureates. The four panels correspond to different choices of the parameters 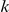 and 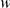. The power law fits (lines) are performed with the maximum likelihood method [26]. The exponents for the direct distribution (of which the cumulative distribution is the integral) are: 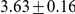 (top left), 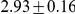 (bottom left), 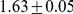 (top right), 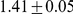 (bottom right). The best fits have the following lower cutoffs and values of the Kolmogorov-Smirnov (KS) statistics: 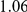, 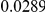 (top left), 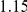, 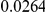 (bottom left), 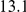, 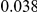 (top right), 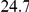, 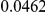 (bottom right). The KS values support the power law ansatz for the shape of the curves. Still, we point out that on the left plots the data span just one decade in the variable, so one has to be careful about the existence of power laws here.

To gain a better understanding of our findings, Figs. 4 and 5 present a statistical analysis of the boosts observed for Nobel Prize Laureates. Figure 4 demonstrates that pronounced peaks are indeed related to highly cited papers. Furthermore, Fig. 5 analyzes the size distribution of peaks. The distribution looks like a power law for all choices of the parameters 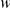 and 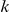 (at least within the relevant range of small values). This suggests that the bursts are produced by citation cascades as they would occur in a self-organized critical system [22]. In fact, power laws were found to result from human interactions also in other contexts [23]–[25].

The mechanism underlying citation cascades is the discovery of new ideas, which colleagues refer to in the references of their papers. Moreover, according to the rich-gets-richer effect, successful papers are more often cited, also to raise their own success. Innovations may even cause scientists to change their research direction or approach. Apparently, such feedback effects can create citation cascades, which are ultimately triggered by landmark papers.

**Figure 6:**
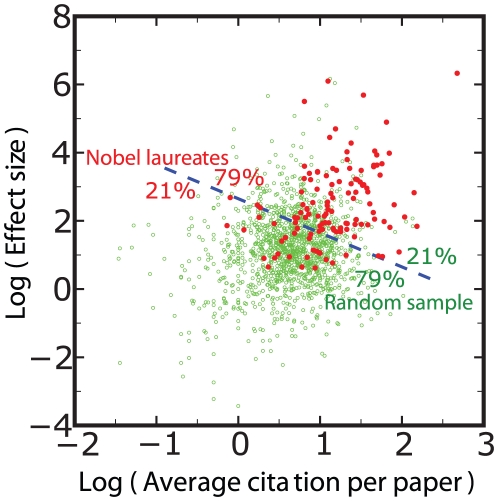
Two-dimensional representation of our collection of Nobel Prize Laureates and a set of 1361 scientists, which were randomly selected. On the x-axis we report the average number of citations of a scientist, on the y-axis his/her boost factor. It can be seen that, on average, Nobel Prize winners clearly perform better. However a Nobel Prize is not solely determined by the average number of citations and the boost factor, but also by further factors. These may be the degree of innovation or quality, which are hard to quantify.

Finally, it is important to check whether the boost factor is able to distinguish exceptional scientists from average ones. Since any criteria used to define “normal scientists” may be questioned, we have assembled a set of scientists taken at random. Scientists were chosen among those who published at least one paper in the year 2000. We selected 400 names for each of four fields: Medicine, Physics, Chemistry and Economy. After discarding those with no citations, we ended up with 1361 scientists. In Fig. 6 we draw on a bidimensional plane each scientist of our random sample (empty circles), together with the Nobel Prize Laureates considered (full circles). The two dimensions are the value of the boost factor and the average number of citations of a scientist. A cluster analysis separates the populations in the proportions of 79% to 21%. The separation is significant but there is an overlap of the two datasets, mainly because of two reasons. First, by picking a large number of scientists at random, as we did, there is a finite probability to choose also outstanding scholars. We have verified that this is the case. Therefore, some of the empty circles deserve to sit on the top-right part of the diagram, like many Nobel Prize Laureates. The second reason is that we are considering scholars from different disciplines, which generally have different citation frequencies. This affects particularly the average number of citations of a scientist, but also the value of the boost factor. In this way, the position in the diagram is affected by the specific research topic, and the distribution of the points in the diagram of Fig. 6 is a superposition of field-specific distributions. Nevertheless, the two datasets, though overlapping, are clearly distinct. Adding further dimensions could considerably improve the result. In this respect, the boost factor can be used together with other measures to better specify the performance of scientists.

## Discussion

In summary, groundbreaking scientific papers have a boosting effect on previous publications of their authors, bringing them to the attention of the scientific community and establishing their “authority”. We have provided the first quantitative characterization of this phenomenon by introducing a new variable, the “boost factor”, which is sensitive to sudden changes in the citation rates. The fact that landmark papers trigger the collective discovery of older papers amplifies their impact and tends to generate pronounced spikes long before the paper receives full recognition. The boosting factor can therefore serve to discover new breakthroughs and talents more quickly than classical citation indices. It may also help to assemble good research teams, which have a pivotal role in modern science [27]–[29].

The power law behavior observed in the distribution of peak sizes suggests that science progresses through phase transitions [30] with citation avalanches on all scales–from small cascades reflecting quasi-continuous scientific progress all the way up to scientific revolutions, which fundamentally change our perception of the world. While this provides new evidence for sudden paradigm shifts [31], our results also give a better idea of why and how they happen.

It is noteworthy that similar feedback effects may determine the social influence of politicians, or prices of stocks and products (and, thereby, the value of companies). In fact, despite the long history of research on these subjects, such phenomena are still not fully understood. There is evidence, however, that the power of a person or the value of a company increase with the level of attention they enjoy. Consequently, our study of scientific impact is likely to shed new light on these scientific puzzles as well.

## Materials and Methods

The basic goal is to improve the signal-to-noise ratio in the citation rates, in order to detect sudden changes in them. An effective method to reduce the influence of papers with largely fluctuating citation rates is to weight highly cited papers more. This can be achieved by raising the number of cites to the power 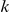, where 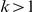. Therefore, our formula to compute 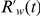 looks as follows:
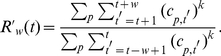
(1)Here, 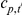 is the number of cites received by paper 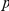 in year 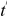. The sum over 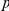 includes all papers published before the year 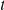; 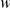 is the time window selected to compute the boosting effect. For 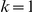 we recover the original definition of 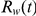 (see main text). For the analysis presented in the paper we have used 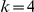 and 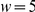, but our conclusions are not very sensitive to the choice of smaller values of 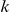 and 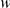.
